# Evaluation of person-centeredness in nursing homes after a palliative care intervention: pre- and post-test experimental design

**DOI:** 10.1186/s12904-019-0431-8

**Published:** 2019-05-31

**Authors:** Christina Bökberg, Lina Behm, Birgitta Wallerstedt, Gerd Ahlström

**Affiliations:** 1 Department of Health Sciences, Faculty of Medicine, Lund University, P.O. Box 157, SE-221 00 Lund, Sweden; 2 Department of Health and Caring Sciences, Faculty of Health and Life Sciences, Centre for Collaborative Palliative Care Linnaeus University, Växjö, Sweden

**Keywords:** Person-centred care, Palliative care, Nursing home, Older persons, Staff education

## Abstract

**Background:**

The needs of care based on palliative principles are stressed for all people with progressive and/or life-limiting conditions, regardless of age and the place in which care is provided. Person-centred palliative care strives to make the whole person visible and prioritizes the satisfaction of spiritual, existential, social, and psychological needs to the same extent as physical needs. However, person-centred palliative care for older persons in nursing homes seems to be sparse, possibly because staff in nursing homes do not have sufficient knowledge, skills, and training in managing symptoms and other aspects of palliative care.

**Methods:**

This study aimed to evaluate whether an educational intervention had any effect on the staff’s perception of providing person-centred palliative care for older persons in nursing homes. Methods: A knowledge-based palliative care intervention consisting of five 2-h seminars during a 6-month period was implemented at 20 nursing homes in Sweden. In total, 365 staff members were participated, 167 in the intervention group and 198 in the control group. Data were collected using two questionnaires, the Person-centred Care Assessment Tool (P-CAT) and the Person-Centred Climate Questionnaire (PCQ-S), answered before (baseline) and 3 months after (follow-up) the educational intervention was completed. Descriptive, comparative, and univariate logistical regression analyses were performed.

**Results:**

Both the intervention group and the control group revealed high median scores in all subscales at baseline, except for the subscale amount of organizational and environmental support in the P-CAT. The staff’s high rating level of person-centred care before the intervention provides limited space for further improvements at follow-up.

**Conclusion:**

This study shows that staff perceived that managers’ and the organization’s amount of support to them in their everyday work was the only area for improvement in order to maintain person-centred care. The experiences among staff are crucial knowledge in understanding how palliative care can be made person-centred in spite of often limited resources in nursing homes. The dose and intensity of education activities of the intervention model need to be tested in future research to develop the most effective implementation model.

**Trial registration:**

NCT02708498. Date of registration 26 February 2016.

**Electronic supplementary material:**

The online version of this article (10.1186/s12904-019-0431-8) contains supplementary material, which is available to authorized users.

## Background

Nursing homes are common sites for older persons (> 65 years) to spend the last part of their lives, [1–3], when they often are suffering from complex and life-limiting conditions [4]. These circumstances require various palliative care needs such as relief from distressing symptoms (pain, anxiety, nausea) to reduce discomfort and promote quality of life until death [1, 5]. Despite these needs, palliative care for older persons in nursing homes seems to be sparse [4, 6]. The approach in both palliative and person-centred care is characterised by a holistic view of the person, and that the person should be supported to live a life with dignity. Person-centred palliative care strives to make the whole person visible and prioritizes the satisfaction of spiritual, existential, social, and psychological needs to the same extent as physical needs [7, 8]. According to Saunders & Kastenbaum [9], a person in a palliative care context is regarded as a human being consisting of physical, mental, social, and spiritual dimensions [9]. Both palliative and person-centred care use partnership and shared decisions based on patient narratives as well as teamwork and documentation of quality of care [8, 10] for proper planning of the person’s remaining life [8].

Palliative care should therefore be provided in a supportive care environment, i.e. a person-centred environment, where the older person and the family feel welcome, seen, and involved by the staff. The concept of a person-centred care environment includes both the physical and psychosocial environment [7, 8]. However, several studies have pointed out that person-centred care is lacking [11–13], although staff perceive that they provide person-centred care [14]. These shortcomings may lead to experiences of unnecessary suffering and decreased quality of life at the final stage of life [4, 5] and suggest an urgent need to improve person-centred palliative care in nursing homes for older persons with chronic illnesses [1, 4, 5, 15].

Traditionally, palliative care has been provided in hospice settings by specialists to persons dying from incurable cancer while persons dying from other diagnoses do not have the same access to palliative care [1, 16]. Yet, the needs of care according to palliative principles are applicable to all people with progressive and/or life-limiting conditions, regardless of age [17] and the place in which care is provided [1].

The multiple morbidities, with complex needs, and symptoms that older persons often have, can make it difficult to identify and handle the last period of life [18, 19]. Therefore, provision of person-centred palliative care for older persons requires both geriatric and palliative expertise. Previous studies found that staff in nursing homes taking care of older persons at the end of life did not talk about death and dying with the residents because of their own fear [20] and uncertainty of existential questions that were perceived as difficult and emotionally demanding tasks for which they lack competence [21]. One possible reason for this is that staff in nursing homes lack education about palliative and end-of-life care [22, 23], and staff do not have sufficient knowledge, skills, and training in managing symptoms and other aspects of palliative care [24–27]. Goddard, et al. [23] reported that staff requested education about palliative care and that they lacked the knowledge to be able to provide appropriate end-of-life-care. This study’s aim was to evaluate whether an educational intervention had any effect on the staff’s perception of providing person-centred palliative care for older persons in nursing homes.

## Methods

### Design

This study is a part of the project “Implementation of Knowledge-based Palliative Care”, abbreviated as the KUPA project from the Swedish name [28]. The KUPA project aimed to improve the person-centeredness of palliative care and the quality of life and participation of older persons in nursing homes and their next of kin in the care process. The KUPA project is designed from an implementation perspective, described in the previous study protocol for the project [28]. In this research approach a successful change in practice, depends on the interplay between several aspects: (1) effectiveness of the implementation strategies; (2) characteristics of the “implementation object”; (3) characteristics of the implementers; (4) the target population; and (5) the context of the implementation [28]. Therefore, the evaluation of the whole KUPA project addresses several different perspectives which will be reported in future papers; staff, managers, context [21], the older persons and their next of kin and also various barriers and facilitating aspects of the implementation [28].

This study, as part of KUPA project, used pre- and post-test experimental design to evaluate staff’s perception of providing person-centred care in both an intervention and control group (clinical trial registration NCT02708498) [28]. The assessment consisted of two questionnaires, one concerning person-centred care and one person-centred climate, answered by staff before and 3 months after (follow-up) the implementation of the 6-month educational intervention, i.e. 9 months from baseline. The same intervals applied to the control nursing homes.

### Setting

There are approximately 2300 nursing homes in Sweden, a country with a population of 10 million people**.** These are accommodations mainly for older persons (> 65 years) who live in their own apartments with care and services provided around the clock. In 2016, approximately 104,000 older persons lived in nursing homes, of those were 4% aged 65 years and 13% aged 80 years or more. Access to an apartment is based upon the older person’s needs and assessed by a social worker in the municipality. Moving into a nursing home usually occurs when the older person is too sick or frail to be able to continue living in their previous home with home care services. The median age in Sweden to move into a nursing home is 86.2 years for women and 83.7 years for men. Most of the residents (67%) are women [29]. The length of stay after moving into nursing homes (time until death) is decreasing [30], and almost one-third of older persons who move into a nursing home die within 6 weeks [4]. Staff at nursing homes mainly consists of nurse assistants, but other professions are also represented, such as registered nurses, physiotherapists, and occupational therapists [31].

### The knowledge-based palliative care intervention

The knowledge-based palliative care intervention consisted of five 2-h educational seminars for staff and frontline managers at nursing homes. The seminars were based on two Swedish national documents on the key principles of palliative care: clinical practice guidelines by the Regional Cancer Centres in co-operation [32] and national guidance on government initiative by the National Board of Health and Welfare [8]. Both documents were based on the WHO definition of palliative care [1, 5, 33] and are intended to improve the quality life for individuals and their families.

An educational booklet, with an uncomplicated language, was designed within the KUPA project to use as study material for staff. The booklet contained five themes: 1) the palliative approach and dignified care, 2) next of kin, 3) existence and dying, 4) symptom relief and 5) collaborative care. The booklet is available in an English version (Additional file 1). The content of the different seminars had a common core for each nursing home and space was left for discussions and questions connected to each of the themes in the booklet. The seminar groups were led by experienced registered nurses and researchers from the field of palliative and geriatric care. The intervention was implemented over a 6-month period during 2016–2017 in two different counties in south of Sweden, in 20 nursing homes. None of the participating nursing homes, have had workplace education or training in palliative care before the KUPA intervention. Eight to twelve staffs were included at each nursing home [28], and the mean score of staff participated in each seminar was 8.

### Sampling and participants

The selection of nursing homes was made through voluntary participation and resulted in a mixture of both larger (more than 100 residents) and smaller nursing homes (less than 25 residents) in the two counties, as well as nursing homes from both urban and rural areas [28].

**Fig. 1:**
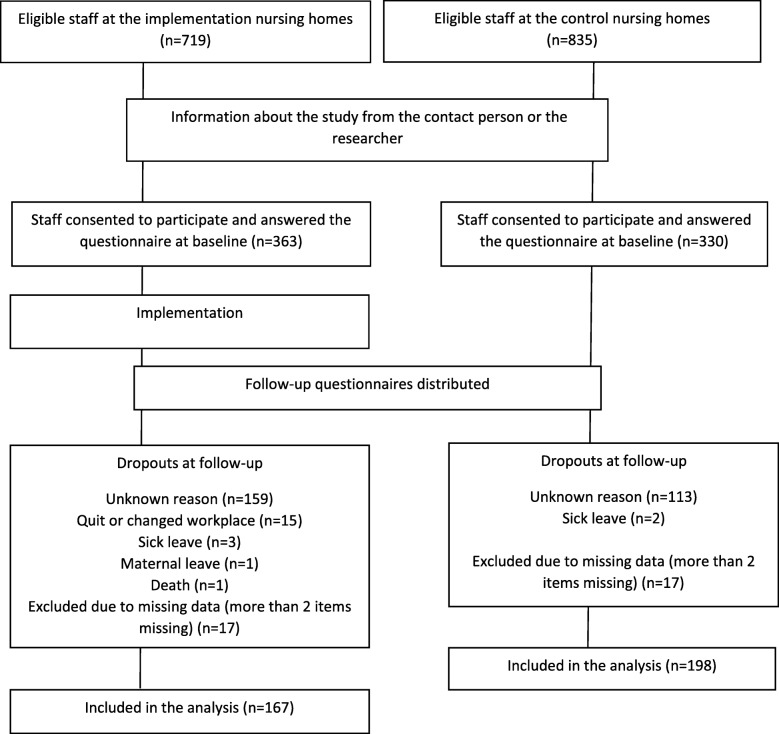
Flow chart showing the inclusion procedure for the study participants

The informants in this study, the staff, were recruited consecutively in equal numbers from both the intervention nursing homes and the control nursing homes. Of 1554 eligible staff, 693 staff consented to participate. Of those consented, 328 staff became drop-out related to declined continued participation, staff quitting or changed workplace, sick leave, maternal leave, or death (*n* = 1). Altogether 365 staff participated, 167 in the intervention group and 198 in the control group (Fig. 1).

In both the intervention group (92%) and the control group (95%), the staff reported experience in general palliative care. In the intervention group, 13% reported experience in specialist palliative care. The corresponding number in the control group was 10% (Table 1).

**Table 1:** Characteristics of the staff in intervention and control group (*n* = 365)

Background variable	Intervention group (n 167)	Control group (n 198)
Age		
median year (range)	47 (21–66)	49 (21–65)
Sex		
Men/Women (number)	10/157	9/189
Working experience		
median year (range)	11 (0–44)	14 (0–41)
Profession (n/%)		
Assistant nurse	151/90	179/90
Registered nurse	8/5	8/4
Occupational therapist	2/1	2/1
Physiotherapist	1/0.5	3/2
Frontline managers	5/3	6/3
Experience in general palliative care		
yes (n/%)	154/92	188/95
Experience in specialist palliative care		
yes (n/%)	21/13	20/10

### Instruments

The data collection was based on two questionnaires, the Person-centred Care Assessment Tool (P-CAT) and the Person-Centred Climate Questionnaire (PCQ-S).

The P-CAT consists of 13 items that measure the extent to which staff rate provided care and service as being person-centred. The instrument exists both in a staff version and a version for older persons. In this study, the version for staff was used. The instrument contains two subscales: 1) *Extent of personalization care*, containing eight items, and 2) *Amount of organizational and environmental support,* containing five items [34]. The first subscale reflects the intentions and actions of the staff to personalize care and prioritize of the interaction as well as provide the possibility for residents to make their own decisions. The second subscale includes factors in the environment and organization that can support or hinder personalization of care and the creation of a positive psychosocial culture. Informants are asked to respond on a five-pointLikert-scale ranging from 1 (disagree completely) to 5 (agree completely). The total score of the scale runs from 13 to 65, with higher values indicating a higher degree of person-centeredness. The Swedish version of the P-CAT has been found to be valid, reliable, and applicable for further use [34, 35]. In this sample, the Cronbach’s alpha estimates were α 0.69 for subscale 1 and α 0.78 for subscale 2.

The PCQ-S consists of 14 items and was used to assess to what extent the climate of the care environments was person-centred. PCQ exists in both a patient (PCQ-P) and a staff version (PCQ-S). In this study, the version for staff was used. PCQ-S has been shown to be a valid and reliable contribution for assessing to what extent the climate of care environments is person-centred [36]. The subscales are 1) *Safety,* consisting of five items, 2) *Everydayness,* consisting of five items, and 3) *Community,* consisting of four items. Informants are asked to respond on a six-pointLikert-scale ranging from 1 (No, I disagree completely) to 6 (Yes, I agree completely). The total score of the scale runs from 14 (a climate that is minimally person-centred) to 84 (a climate that is maximally person-centred). In this sample, the Cronbach’s alpha estimates were α 0.85 for subscale 1, α 0.90 for subscale 2, and α 0.86 for subscale 3.

### Data collection

Before the start of the study, the researcher and the manager of the nursing homes informed the staff of the aim of the study and details of their participation. Then, a contact person, usually the frontline manager, was chosen at each nursing home. The contact person was responsible for the contact between the researcher and the staff. The researchers informed the contact person about the study. In some of the nursing homes, the contact person informed the staff about the study and in what way the staff would be involved, and in other nursing homes, the researchers were invited to present the study at regular meetings. It was stressed that participation was voluntary. Dates for distributing the questionnaires were decided in advance (with 6 months in between time points). The contact person distributed the questionnaires to the staff at the decided time. The inclusion criteria were having a permanent position at the nursing home as an assistant nurse, registered nurse, physiotherapist, occupational therapist, social worker, or unit manager. The staff invited to participate received written information, a consent form, and the questionnaires. Locked mailboxes, one for the consent and one for the questionnaire, were placed at each nursing home. Based upon the response frequencies from the staff at each nursing home, the researcher provided several (up to 3) reminders to the contact person at both the baseline and follow-up.

### Statistical analyses

The questionnaire data were analysed using methods applicable for within-group comparisons (data after intervention compared with data before in the intervention group and the control group). The selection of methods aside from descriptive statistics was based on whether the data were distributed normally and the scale level of the instruments. The Wilcoxon signed rank test was used to compare paired data within the groups. Furthermore, subgroup analyses within the intervention group to explore differences regarding between those who participated in the intervention and those who did not was performed. Either the Pearson chi-square test or Fisher’s exact test (if any expected cell value was less than 5) was used. The Mann-WhitneyU-test and the Kruskal-Wallis test was applied to compare the baseline characteristics of the two groups.

Missing data on single items were replaced by the mean score for that item for the appropriate group [35]. Missing data of more than two items the questionnaire was excluded. To evaluate whether there were any differences in improvement between the study arms, the number of participants that had improved scores on the P-CAT and PCQ-S was compared at baseline and follow-up after intervention. In the final analysis, the participants were dichotomized into improved/not improved (same/or lower scores) from baseline to follow-up. A univariate logistic regression analysis was performed with the independent variable improvement/non-improvement in P-CAT and PCQ-S and the dependent variable group (control/implementation). Changes were regarded as statistically significant if the two-tailed*p* value was < 0.05. Analyses were performed using IBM SPSS Statistics version 24.

## Results

No statistically significant differences were detected between the two groups at baseline. The result indicates that the care and service provided at the nursing homes before the intervention had a person-centred approach with little room for improvement. All subscales at baseline in both the intervention group and the control group revealed high median scores, except for the subscale “Amount of organizational and environmental support” (P-CAT). This subscale includes the five items “I simply do not have the time to provide person-centred care”, “The environment feels chaotic”, “We have to get the work done before we can worry about a homelike environment”, “The organization prevents me from providing person-centred care” and “It is hard for residents in the facility to find their way around”.

### Pre- and post-asessment of P-CAT

The overall results of the P-CAT showed no statistically significant changes in nursing home settings concerning person-centeredness after the KUPA intervention in either of the two subscales *Extent of personalizing care* and *Amount of organizational and environmental support* (Table 2). This result was the same when exploring differences between the staff that participated in the intervention with those who did not.

**Table 2:** Intervention group (*n* = 167) and control group (*n* = 198) analyses before and after intervention

Scale and subscale	Intervention group Baseline Median (Q1-Q3)	Intervention group Follow-up Median (Q1-Q3)	Significance p-value^2^	Control group Baseline Median (Q1-Q3)	Control group Follow-up Median (Q1-Q3)	Significance p-value^2^
**P-CAT** ^**3**^						
Total (13–65)	45.0 (41.0–48.0)	45.0 (42.0–48.0)	0.715	44.0 (41.0–48.0)	44.0 (41.0–48.0)	0.601
Extent of personalizing care(8–40^1^)	34. 0 (32.0–38.0)	34.0 (32.0–37.0)	0.837	33.0 (31.0–36.0)	34.0 (31.0–36.0)	0.417
Amount of organizational and environmental support(5–25^1^)	11.0 (7.0–14.0)	11.0 (8.0–14.0)	0. 479	11.0 (8.0–15.0)	11.0 (8.0–15.0)	0.237
**PCQ-S** ^**4**^						
Total (14–84)	77.0 (68.0–82.0)	77.0 (68.0–81.0)	0.685	71.0 (61.0–80.0)	74.0 (67.0–79.0)	0.451
Safety(5–30^1^)	28.0 (25.0–30.0)	27.0 (25.0–30.0)	0.942	27.0 (25.0–29.0)	27.0 (24.0–29.0)	0.693
Everydayness(5–30^1^)	26.0 (22.0–29.0)	25.0 (22.0–29.0)	0.494	24.0 (21.0–28.0)	25.0 (21.0–28.0)	0.374
Community(4–24^1^)	23.0 (21.0–24.0)	23.0 (20.0–24.0)	0.280	22.0 (22.0–24.0)	22.0 (21.0–24.0)	0.051

^1^Underlined score is the most favourable score. ^2^ Wilcoxon Signed Rank Test. Q1 = first quartile; Q3 = third quartile. Significant values are bold. ^3^Missing *n* = 10 in intervention group and *n* = 17 in control group. ^4^Missing *n* = 22 in intervention group and *n* = 33 in control group

No differences in improvement were detected regarding person-centred care (P-CAT) compared to baseline. In Table 3, the proportion n (%), the odds ratio (OR), the 95% confidence interval (CI), and *p*-value for improvement from baseline to follow up in staff’s perceptions of person-centred care and person-centred care climate between the study arms in the KUPA project are presented.

**Table 3:** Improvement from baseline to follow-up between the study arms

Outcome measure	Intervention group (*n* = 167)			Control group (*n* = 198)		
	n (%)	OR	CI	n (%)	OR	p-value
P-CAT^1^						
Extent of personalizing care	69 (44)	1.115	0.734–1.695	76 (42)	1	0.717
Amount of organizational and environmental support	75 (48)	1.406	0.926–2.135	72 (40)	1	0.108
PCQ-S^2^						
Safety	54 (37)	0.828	0.490–1.400	66 (40)	1	0.482
Everydayness	52 (36)	0.594	0.343–1.030	74 (45)	1	0.064
Community	57 (39)	1.081	0.068–1.861	66 (40)	1	0.778

^1^Missing *n* = 10 in intervention group and *n* = 17 in control group. ^2^Missing *n* = 22 in intervention group and *n* = 33 in control group

### Pre- and post-intervention assessment of PCQ-S

PCQ-S was used to measure changes in the climate of the nursing homes settings concerning person-centeredness after implementation of the educational intervention. The results of the control group revealed close to a statistically significant decline (p 0.051) on the subscale *Community*. No statistically significant changes in the intervention group were detected for any of the three subscales (Table 2). This result was the same when exploring differences between the staff that participated in the intervention with those who did not.

No differences in improvement regarding person-centred care climate (PCQ-S) compared to baseline were detected (Table 3).

## Discussion

This study aimed to evaluate whether an educational palliative care intervention for staff and frontline managers had any effect on their perception of providing person-centred care for older persons in nursing homes. Previous attempts at palliative care interventions at nursing homes are sparse, and, as far as we know, no study has previously used person-centred care as an outcome for evaluating a palliative care intervention even though person-centred care is a core concept in palliative care.

Our results showed no improvement in any of the outcomes (P-CAT or PCQ-S). This might imply that the intervention was not successful. The fact that our intervention did not focus on person-centred care explicitly might be one reason for the lack of improvement in the intervention group. However, there may be several other explanations for our results. One reason could be that the participants did not practice the knowledge learned from the intervention and this was an obstacle for disseminate knowledge about the intervention to their colleagues that not participated in the education seminars. One benefit with workplace training is the easy way of dissemination of knowledge to colleagues in comparison with course or conference education for one or a few persons at external location. Another reason could be the relationship between person-centred care and palliative care. Both person-centred care and palliative care are ambiguous concepts without a distinct consensus [37]. It has also been claimed that the ideal of person-centred care has not been integrated explicitly in palliative care [38] and that this integration seems to be even more challenged due to increasing needs for palliative care globally [37, 39]. However, Lavoie, Blondeau, & Martineau [40] found that integration of a person-centred approach in palliative care contributed to a change where the care focus moved from being task-centred to being person-centred. According to Grassi [41], a person-centred way of communication facilitates the ill person’s understanding of the actual situation and the transition to palliative care. However, Öhlén et al. [37] argued that person-centred care is an ethical stance in palliative care.

Comparing our P-CAT results to other studies, staff at the included nursing homes in southern Sweden rate the person-centeredness in care similar to other studies in a nursing home context in Sweden [34] and in Norway, [42–44]. However, our rate was higher compared to nursing homes for persons with dementia in Sweden [45]. When comparing the results on person-centred climate (PCQ-S), our study showed high ratings compared to other Swedish studies in nursing homes [46], and nursing homes for persons with dementia [45].

A review of the literature [47] showed promising results of palliative care interventions on satisfaction with care, lower observed discomfort in residents with end-stage dementia, more referrals to hospice services, fewer hospital admissions and days in hospital and an increase in do-not-resuscitate orders and documented advance care plan discussions. However, Froggatt [48] emphasized that a cultural change is needed to bring sufficient changes in practice at nursing homes to achieve evidence-based palliative care and that education alone is not sufficient. The intervention in our study was designed as a palliative education in five seminars. The dose of the educational intervention, during the implementation period of 6 months, was probably too small to create a change in the care culture and may have contributed to the lack of improvement. The importance of culture was evident in other studies [49–51], but philosophy and organization of care also governed staff approach to end-of-life care. This is in agreement with our results that staff reported low person-centred care in respect to the P-CAT scale “Amount of organizational and environmental support”.

Studies have shown that facilitators of successful implementation of a culture change are good leadership, strong teamwork and efficient communication systems [52, 53]. A study of the implementation of person-centred care in nursing homes [54] found that effective implementation is associated with a confident leadership communication about the goals for change and the organizational readiness for change of the care providers.

A study conducted within the KUPA project explored the readiness to implement palliative care in the included nursing homes from the perspective of managers [55] and found both barriers to and facilitators for implementation. One of the barriers was fear or hesitation among the staff to face dying persons and to talk to them about existential issues. An ethnographic study in the KUPA project verified that conversations about death and dying was related to obstacles among assistant nurses [21]. To be able to talk about preferences in the end of life, to get to know the older person and create a close relationship has been highlighted as a key aspect to providing quality end-of-life care [51]. In person-centred care, it is also necessary to understand the behaviours and psychological symptoms from the perspective of the person [7]. The person and the staff need to share information and the persons should be able to participate on equal basis in decisions concerning care and treatment [7], and the fear of death and dying thus hampers the implementation of care.

Another well-known barrier to the implementation of palliative care in nursing homes described by Nilsen et al. [55] and in other studies with the aim to improve palliative care in nursing homes [51, 56, 57] is resources and time. Limited resources restrict the number of staffs per older person, which has an impact on the time available for communication with the older persons, their families, and colleagues, which is crucial to be able to perform person-centred care. The time the older persons spend at the nursing homes in Sweden is decreasing [4, 29] because they are frailer and often have multi-morbidity and complex care needs [17] when they move there. However, it is not obvious that more staff are employed to manage the increased workload. Instead, the staff must learn and include new initiatives and/or recommendations from the organization in their practice. These initiatives are often additive to the staff’s already heavy workload and might result in change fatigue. Change fatigue is the overwhelming feeling of stress, exhaustion, and burnout associated with rapid and continuous change in the workplace [58, 59]. Frequent changes in the organization can also cause a saturation effect, which occurs when there is no period of recovery between the changes in the organization [60]. Nilsen et al. [55] reported that the managers stressed that the staff were tired of changes, which made them less interested in acquiring new knowledge and skills required to develop person-centred palliative care.

Another limitation of the study is the drop-out rate due to declined continued participation, staff quitting or changed workplace, sick leave, maternal leave energy was considerable (47%) and higher than expected, leaving us with a limited number of staff (n 365) included in the analytical part. The lessons learned from this study is to test intervention with a higher dose of training through more frequent occasions during a longer period than 6 months and provide the education to all staff at the participated nursing homes.

In a narrative review, 24 studies from three countries [61] were compared in terms of person-centred interventions, measurement, and resident outcomes. One conclusion drawn from the study is that the concept of person-centred care is ambiguous, with no agreement upon definition, which is crucial when developing and implementing this care model. According to Ekman et al. [14], it can be difficult for staff to always put the person before the disease even if they apply person-centred care, which can be important when evaluating the result of a palliative care intervention according to person-centeredness. However, even if person-centred care has been an increasing focus in Sweden during the last years, a paradigm shift takes time and is not done overnight [14].

There is a known gap between what we know and what we do in practice in health-care [62], which has been the rationale to implement evidenced-based palliative knowledge through the KUPA project. In the ethnographic study [21] within the project concerning how nurse assistants performed existential conversations with residents at nursing homes, this gap was highlighted. The nurse assistants had tools and knowledge of how to handle questions and thoughts from the residents about dying and death, but in practice, they lacked time, and emotional strain was another obstacle preventing them from using their knowledge. Gladman et al. [62] highlighted the importance of organizational direction to get professional training, which may generate professionals with interest in and skills to transform knowledge into practice. They also proposed a collaborative non-linear, multidirectional, and iterative process of knowledge exchange that involve staff at nursing homes, not the traditional model where researchers present evidence that should be implemented in clinical practice. Therefore, the educational intervention in KUPA project was designed as a training for staff and frontline managers consisting of evidence-based knowledge in a booklet constructed for the project and reflective discussions in five seminars as well as assignments to do in preparation before each seminar or to complete after each seminar. The assignments inspired the staff to take “cases” from their daily work. A list of references for further self-study by the staff was also given in the booklet (Additional file 1). Rowley, Morris, Currie, and Schneider [63] have described attempts to bridge the gap between research and practice by having researchers involved in both research and clinical practice. Even Cooke, Langley, Wolstenholme, and Hampshaw [64] highlighted the importance of research as a result from co-production, but this requires time and resources to overcome boundaries and promoting leaders.

## Conclusions

This study evaluated the staff’s perception of providing person-centred palliative care for older persons in nursing homes after they participated in an educational palliative care intervention. The results indicated that the care and service provided at the nursing homes before the intervention had a person-centred approach, and the only perceived improvement area in person-centred care is the managers’ and organization’s support of the staff’s everyday work in order to maintain person-centred care. The lessons learned from the study are to educate all staff at the participated nursing homes, more frequent training and use of a longer period than half a year and specifically educate some staff at each nursing home to educate newly employed staff on the person-centred palliative care approach. Not only staff needs education, but also front leaders, since the study found lack of leadership and embedded organisational support for person-centred care. Further research could focus on investigating front leaders’ support to staff and removing obstacles for providing person-centred care.

## Additional file

Additional file 1: The Booklet Themed meetings about palliative care within health care. (PDF 610 kb)

## Data Availability

Even though the data are anonymized, the study contains sufficient details to enable identification of individuals. Therefore, before approving the study, the Regional Ethics Review Board in Lund set severe restrictions regarding the accessibility of the data, but they are available from the project leader (GA) on reasonable request. Written permission to share or distribute the booklet can be received by Associate Professor Anna Sandgren, Center for Collaborative Palliative Care, Linnaeus University, Sweden. E-mail: anna.sandgren@lnu.se Most Photos AB has granted permission to use the image on the first page to Gerd Ahlström.

## Abbreviations

CI: Confidence interval
KUPA project: Implementation of Knowledge-based Palliative Care
OR: Odds ratio
P-CAT: Person-centred Care Assessment Tool
PCQ-S: Person-Centred Climate Questionnaire for staff
WHO: World Health Organization

## References

[1] Hall S, Petkova H, Tsouros A, Constantini M, Higginson I (2011). Palliative care for older people: better practices. Copenhagen: WHO regional Office for Europe.

[2] Houttekier Dirk, Cohen Joachim, Bilsen Johan, Addington-Hall Julia, Onwuteaka-Philipsen Bregje D., Deliens Luc (2010). Place of Death of Older Persons with Dementia. A Study in Five European Countries. Journal of the American Geriatrics Society.

[3] Håkanson Cecilia, Öhlén Joakim, Morin Lucas, Cohen Joachim (2015). A population-level study of place of death and associated factors in Sweden. Scandinavian Journal of Public Health.

[4] Smedbäck Jonas, Öhlén Joakim, Årestedt Kristofer, Alvariza Anette, Fürst Carl-Johan, Håkanson Cecilia (2017). Palliative care during the final week of life of older people in nursing homes: A register-based study. Palliative and Supportive Care.

[5] Davies E, Higginson IJ (2004). Better palliative care for older people.

[6] Seymour Jane (2011). Changing times: preparing to meet palliative needs in the 21st Century. British Journal of Community Nursing.

[7] McCormack Brendan, McCance Tanya V. (2006). Development of a framework for person-centred nursing. Journal of Advanced Nursing.

[8] National Board of Health and Welfare. The national knowledge support document for good palliative care at the end of life. [in Swedish: Nationellt kunskapsstöd för god palliativ vård i livets slutskede – Vägledning, Rekommendationer och indikatorer – Stöd för styrning och ledning]. Stockholm: Sweden. 2013. Accessed 4 Jul 2018. https://www.socialstyrelsen.se/Lists/Artikelkatalog/Attachments/19107/2013-6-4.pdf

[9] Saunders CM, Kastenbaum R (1997). Hospice care on the international scene.

[10] World Health Organization. National cancer control programs: policies and managerial guidelines. 2nd ed. Geneva; 2002. https://apps.who.int/iris/bitstream/handle/10665/42494/9241545577.pdf;jsessionid=E769D128437799DF1EB646328ECE425F?sequence=1. Accessed 22 May 2019.

[11] Dewing Jan (2004). Concerns relating to the application of frameworks to promote person-centredness in nursing with older people. Journal of Clinical Nursing.

[12] McCormack Brendan (2003). A conceptual framework for person-centred practice with older people. International Journal of Nursing Practice.

[13] McCormack Brendan, Karlsson Bengt, Dewing Jan, Lerdal Anners (2010). Exploring person-centredness: a qualitative meta-synthesis of four studies. Scandinavian Journal of Caring Sciences.

[14] Ekman Inger, Swedberg Karl, Taft Charles, Lindseth Anders, Norberg Astrid, Brink Eva, Carlsson Jane, Dahlin-Ivanoff Synneve, Johansson Inga-Lill, Kjellgren Karin, Lidén Eva, Öhlén Joakim, Olsson Lars-Eric, Rosén Henrik, Rydmark Martin, Sunnerhagen Katharina Stibrant (2011). Person-Centered Care — Ready for Prime Time. European Journal of Cardiovascular Nursing.

[15] Froggatt Katherine, Payne Sheila, Morbey Hazel, Edwards Michaela, Finne-Soveri Harriet, Gambassi Giovanni, Pasman H. Roeline, Szczerbińska Katarzyna, Van den Block Lieve (2017). Palliative Care Development in European Care Homes and Nursing Homes: Application of a Typology of Implementation. Journal of the American Medical Directors Association.

[16] Brännström Margareta, Hägglund Lena, Fürst Carl Johan, Boman Kurt (2011). Unequal care for dying patients in Sweden: a comparative registry study of deaths from heart disease and cancer. European Journal of Cardiovascular Nursing.

[17] Wowchuk Suzanne M, McClement Susan, Bond Jr John (2007). The challenge of providing palliative care in the nursing home. International Journal of Palliative Nursing.

[18] Handley Melanie, Goodman Claire, Froggatt Katherine, Mathie Elspeth, Gage Heather, Manthorpe Jill, Barclay Stephen, Crang Clare, Iliffe Steve (2013). Living and dying: responsibility for end-of-life care in care homes without on-site nursing provision - a prospective study. Health & Social Care in the Community.

[19] Johnson Martin, Attree Moira, Jones Ian, Al Gamal Ekhlas, Garbutt David (2013). Diagnosis, prognosis and awareness of dying in nursing homes: towards the Gold Standard?. International Journal of Older People Nursing.

[20] Österlind Jane, Ternestedt Britt-Marie, Hansebo Görel, Hellström Ingrid (2016). Feeling lonely in an unfamiliar place: older people’s experiences of life close to death in a nursing home. International Journal of Older People Nursing.

[21] Alftberg Åsa, Ahlström Gerd, Nilsen Per, Behm Lina, Sandgren Anna, Benzein Eva, Wallerstedt Birgitta, Rasmussen Birgit (2018). Conversations about Death and Dying with Older People: An Ethnographic Study in Nursing Homes. Healthcare.

[22] Fryer S, Bellamy G, Morgan T, Gott M. Sometimes I’ve gone home feeling that my voice hasn’t been heard, a focus group study exploring the views and experiences of health care assistants when caring for dying residents. BMC Palliative Care. 2016. 10.1186/s12904-016-0150-3. 10.1186/s12904-016-0150-3 PMC4992208 27543042

[23] Goddard Cassie, Stewart Frances, Thompson Genevieve, Hall Sue (2011). Providing End-of-Life Care in Care Homes for Older People. Journal of Applied Gerontology.

[24] Brazil Kevin, Kaasalainen Sharon, McAiney Carrie, Brink Peter, Kelly Mary Lou (2012). Knowledge and perceived competence among nurses caring for the dying in long-term care homes. International Journal of Palliative Nursing.

[25] Bruera Eduardo, Willey Jie S., Ewert-Flannagan Patricia A., Cline Mary K., Kaur Guddi, Shen Loren, Zhang Tao, Palmer J. Lynn (2004). Pain intensity assessment by bedside nurses and palliative care consultants: a retrospective study. Supportive Care in Cancer.

[26] Levine S, O’Malley S, Baron A, Ansari A, Deamant C, Frader J, et al. Training the workforce: description of a longitudinal interdisciplinary education and mentoring program in palliative care. J Pain Symptom Manag. 2017. 10.1016/j.jpainsymman. 10.1016/j.jpainsymman.2016.11.009 28062351

[27] Andersson Sofia, Årestedt Kristofer, Lindqvist Olav, Fürst Carl-Johan, Brännström Margareta (2018). Factors Associated With Symptom Relief in End-of-Life Care in Residential Care Homes: A National Register-Based Study. Journal of Pain and Symptom Management.

[28] Ahlström G, Nilsen P, Benzein E, Behm L, Wallerstedt B, Persson M, Sandgren A. Implementation of knowledge-based palliative care in nursing homes and pre-post evaluation by cross-over design: a study protocol. BMC Palliative Care. 2018. 10.1186/s12904-018-0308-2. 10.1186/s12904-018-0308-2 PMC5863832 29566688

[29] National board of health and welfare. Statistik om särskilt Boende (statistics for nursing homes). [in Swedish]. 2016a. Accessed 4 Jul 2018. http://www.socialstyrelsen.se/Lists/Artikelkatalog/Attachments/20404/2016-12-5.pdf

[30] Schön Pär, Lagergren Mårten, Kåreholt Ingemar (2015). Rapid decrease in length of stay in institutional care for older people in Sweden between 2006 and 2012: results from a population-based study. Health & Social Care in the Community.

[31] National Board of Health and Welfare Vård och omsorg om äldre. Lägesrapport 2016. (care and service for elderly. Progress report 2016). [in Swedish]. (2016b). Accessed 4 Jul 2018. https://www.socialstyrelsen.se/Lists/Artikelkatalog/Attachments/20088/2016-2-29.pdf

[32] Regional Cancer Centres in co-operation (Regionala cancercentrum i samverkan). The national care program for palliative care 2012–2014. [in Swedish]. 2012.

[33] Connor R, Sepulveda Bermedo MC. Global atlas of palliative Care at the end of life. Worldwide palliative care Alliance. 2014. Accessed 11 June 2018. http://www.who.int/nmh/Global_Atlas_of_Palliative_Care.pdf

[34] Sjögren Karin, Lindkvist Marie, Sandman Per-Olof, Zingmark Karin, Edvardsson David (2011). Psychometric evaluation of the Swedish version of the Person-Centered Care Assessment Tool (P-CAT). International Psychogeriatrics.

[35] Edvardsson David, Fetherstonhaugh Deirdre, Nay Rhonda, Gibson Stephen (2009). Development and initial testing of the Person-centered Care Assessment Tool (P-CAT). International Psychogeriatrics.

[36] EDVARDSSON DAVID, SANDMAN P.O., RASMUSSEN BIRGIT (2009). Construction and psychometric evaluation of the Swedish language Person-centred Climate Questionnaire - staff version. Journal of Nursing Management.

[37] Öhlén Joakim, Reimer-Kirkham Sheryl, Astle Barbara, Håkanson Cecilia, Lee Joyce, Eriksson Marjukka, Sawatzky Richard (2017). Person-centred care dialectics-Inquired in the context of palliative care. Nursing Philosophy.

[38] Cherny NI, Fallon MT, Kaasa S, Portenoy RK, Currow DC (2015). Oxford textbook of palliative medicine.

[39] Seymour Jane E, Kumar Arun, Froggatt Katherine (2011). Do nursing homes for older people have the support they need to provide end-of-life care? A mixed methods enquiry in England. Palliative Medicine.

[40] Lavoie Mireille, Blondeau Danielle, Martineau Isabelle (2013). The integration of a person-centered approach in palliative care. Palliative and Supportive Care.

[41] Grassi Luigi (2015). Communicating anticancer treatment cessation and transition to palliative care: The need for a comprehensive and culturally relevant, person-centered approach. Cancer.

[42] Rokstad Anne Marie Mork, Engedal Knut, Edvardsson David, Selbaek Geir (2012). Psychometric evaluation of the Norwegian version of the Person-centred Care Assessment Tool. International Journal of Nursing Practice.

[43] Jacobsen FF, Mekki TE, Førland O, Folkestad B, Kirkevold Ø, Skår R, et al. A mixed method study of an education intervention to reduce use of restraint and implement person-centered dementia care in nursing homes. BMC Nurs. 2017. 10.1186/s12912-017-0244-0. 10.1186/s12912-017-0244-0 PMC5604397 28936121

[44] Røen Irene, Kirkevold Øyvind, Testad Ingelin, Selbæk Geir, Engedal Knut, Bergh Sverre (2017). Person-centered care in Norwegian nursing homes and its relation to organizational factors and staff characteristics: a cross-sectional survey. International Psychogeriatrics.

[45] Edvardsson David, Sandman P. O., Borell Lena (2014). Implementing national guidelines for person-centered care of people with dementia in residential aged care: effects on perceived person-centeredness, staff strain, and stress of conscience. International Psychogeriatrics.

[46] Edvardsson David, Sjögren Karin, Lindkvist Marie, Taylor Michael, Edvardsson Kristina, Sandman P.O. (2013). Person-centred climate questionnaire (PCQ-S): establishing reliability and cut-off scores in residential aged care. Journal of Nursing Management.

[47] Hall S, Kolliakou A, Petkova H, Froggatt K, Higginson IJ. Interventions for improving palliative care for older people living in nursing care homes. Cochrane Database Syst Rev. 2011. 10.1002/14651858.CD007132.pub2. 10.1002/14651858.CD007132.pub2 PMC6494579 21412898

[48] Froggatt Katherine A (2001). Palliative care and nursing homes: where next?. Palliative Medicine.

[49] Casey Dympna, Murphy Kathy, Ni Leime Aine, Larkin Philip, Payne Sheila, Froggatt Katherine A, O’Shea Eamon (2011). Dying well: factors that influence the provision of good end-of-life care for older people in acute and long-stay care settings in Ireland. Journal of Clinical Nursing.

[50] Clarke Amanda, Ross Helen (2006). Influences on nurses’ communications with older people at the end of life: perceptions and experiences of nurses working in palliative care and general medicine. International Journal of Older People Nursing.

[51] Hopkinson Jane B., Hallett Christine E., Luker Karen A. (2003). Caring for dying people in hospital. Journal of Advanced Nursing.

[52] Barba BE, Tesh AS, Courts NF (2002). Promoting thriving in nursing homes: the Eden alternative. J Gerontol Nurs.

[53] Steiner JL, Eppelheimer C, De Vries M (2004). Successful Edenization through education: suggestions for encouraging LTC staff to embrace the change of the Eden alternative. Nurs Homes.

[54] Rosemond Cherie A., Hanson Laura C., Ennett Susan T., Schenck Anna P., Weiner Bryan J. (2012). Implementing person-centered care in nursing homes. Health Care Management Review.

[55] Nilsen P, Wallerstedt B, Behm L, Ahlström G. Towards evidence-based palliative care in nursing homes in Sweden: a qualitative study informed by the organizational readiness to change theory. Implement Sci. 2018. 10.1186/s13012-017-0699-0. 10.1186/s13012-017-0699-0 PMC5753464 29301543

[56] Brueckner T, Schumacher M, Schneider N. Palliative care for older people—exploring the views of doctors and nurses from different fields in Germany. BMC Palliat Care. 2009. 10.1186/1472-684X-8-7. 10.1186/1472-684X-8-7 PMC2706814 19549336

[57] Lynch T., Clark D., Centeno C., Rocafort J., de Lima L., Filbet M., Hegedus K., Belle O., Giordano A., Guillén F., Wright M. (2010). Barriers to the development of palliative care in Western Europe. Palliative Medicine.

[58] Bernerth Jeremy B., Walker H. Jack, Harris Stanley G. (2011). Change fatigue: Development and initial validation of a new measure. Work & Stress.

[59] Brown Robin, Wey Howard, Foland Kay (2018). The Relationship Among Change Fatigue, Resilience, and Job Satisfaction of Hospital Staff Nurses. Journal of Nursing Scholarship.

[60] Ead Heather (2015). Change Fatigue in Health Care Professionals—An Issue of Workload or Human Factors Engineering?. Journal of PeriAnesthesia Nursing.

[61] Li Junxin, Porock Davina (2014). Resident outcomes of person-centered care in long-term care: A narrative review of interventional research. International Journal of Nursing Studies.

[62] Gladman John R. F., Conroy Simon Paul, Ranhoff Anette Hylen, Gordon Adam Lee (2016). New horizons in the implementation and research of comprehensive geriatric assessment: knowing, doing and the ‘know-do’ gap. Age and Ageing.

[63] Rowley E, Morriss R, Currie G, Schneider J. Research into practice: collaboration for leadership in applied Health Research and care (CLAHRC) for Nottinghamshire, Derbyshire, Lincolnshire (NDL). Implement Sci. 2012. 10.1186/1748-5908-7-40. 10.1186/1748-5908-7-40 PMC3441357 22553966

[64] Cooke Jo, Langley Joe, Wolstenholme Dan, Hampshaw Susan (2016). "Seeing" the Difference: The Importance of Visibility and Action as a Mark of "Authenticity" in Co-production Comment on "Collaboration and Co-production of Knowledge in Healthcare: Opportunities and Challenges". International Journal of Health Policy and Management.

[65] World Medical Association (2013). WMA Declaration of Helsinki – Ethical principles for medical research involving human subjects.

[66] SFS 2003:460. Lag om etikprövning av forskning som avser människor. (Act concerning the ethical review of research involving humans). Stockholm, ministry of education and research; 2003.

[67] Board EDP (2018). the General Data Protection Regulation GDPR.

