# Supplementary material for: Evaluation of person-centeredness in nursing homes after a palliative care intervention: pre- and post-test experimental design
Source: BMC Palliat Care. 2019 May 31;18:44. doi: 10.1186/s12904-019-0431-8 (PMC6543575; doi:10.1186/s12904-019-0431-8)
Supplement: Supplementary file 1 — The Booklet Themed meetings about palliative care within health care. (PDF 610 kb) [file 12904_2019_431_MOESM1_ESM.pdf]

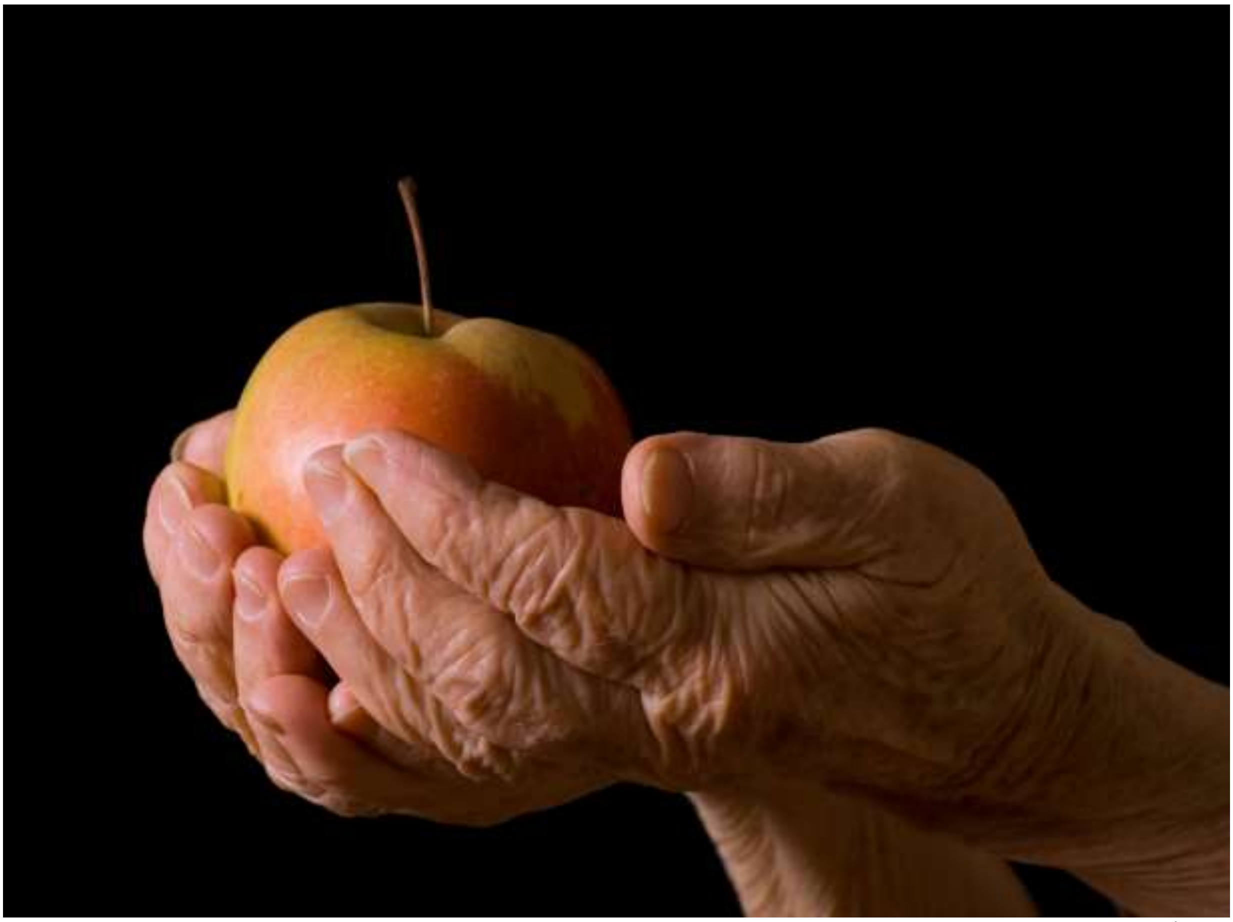

Photo© Mostphotos AB. www.mostphotos.com/sv-se

# Themed meetings about palliative care within health care

**© Swedish version: Center for Collaborative Palliative Care, Linnaeus University, Sweden.**
**Permission to translate into English and to use given by Professor Eva Benzein, Linnaeus University, Sweden. English translation by Malcolm Forbes. © English version: Eva Benzein, Center for Collaborative Palliative Care, Linnaeus University, Sweden and Gerd Ahlström, Department of Health Sciences, Lund University.**

Please note that some of the references in the Swedish version has been replaced with English language equivalents in the English version of the booklet.

# Contents


Welcome to themed meetings within palliative care 4

- Themed meetings 5
- Material for the meetings 5
- The work of improvement 7
- Selected references in English 8

The palliative approach and dignified care 9

- Palliative care 9
- The WHO definition of palliative care 10
- The values underlying palliative care 10
- The transition to palliative care 11
- Dignified care — preservation of the patient's dignity 12
- Dignified care — a case history 13
- Non-dignified care — a case history 13
- As follow-up... 14
- The rights of the dying 14
- References 15
- Suggestions for further reading 15

Next of kin 16

- The situation of the next of kin 16
- Children as next of kin 17
- Participation 18
- Support 18
- As follow-up 19
- References 20
- Suggestions for further reading 20

Existence and dying 21

- Existence 21
- Dying 23
- The place where care is given 23
- The dead body 24
- As follow-up 25
- References 25
- Suggestions for further reading 25

The alleviation of symptoms 26
Symptoms 26
The assessment of symptoms 27
Examples of instruments for the assessment of symptoms 27
General symptom assessment 28
Specific symptom assessment 28
The alleviation of symptoms 28
As follow-up 29
References 29
Suggestions for further reading 29

Collaborative care 30
The concept of collaboration in care 30
Participation and self-determination 31
The prerequisites for collaborative care 32
The act of collaboration 32
As follow-up 33
References 33
Suggestions for further reading 33

References in English 34

Questions about the Swedish booklet can be answered by Associate Professor Anna Sandgren, Linnaeus University and of the translated English booklet by Professor Gerd Ahlström, Lund University.

# Welcome to themed meetings within palliative care

The original version is developed by the Center for Collaborative Palliative Care, Linnæus University, Sweden which has given permission into English and to be used by Lund University. The purpose of the meetings is to offer the participants the opportunity for acquiring knowledge and inspiration that they can afterwards put to use in their work.

The content in the material for the meetings is based on the WHO documents, *Better Palliative Care for Older People* (2004), (hereinafter referred to as the WHO 2004) and *Palliative Care for Older People: Better Practices* (2011), (hereinafter referred to as the WHO 2011) as well as the Swedish *National Care Programme for Palliative Care 2012–14* (hereinafter referred to as the NCP, only in Swedish) and the Swedish *National Knowledge Support for Good Palliative Care in Life's Final Phase* (hereinafter referred to as the NKS, only in Swedish). These documents are directed towards all professions concerned with palliative care and are designed to serve as a basis for the dignified care of persons who are gravely ill or dying and for the welfare of their next of kin. The themes covered are the following:

- The palliative approach and dignified care
- Next of kin
- Existence and dying
- The alleviation of symptoms
- Collaborative care

# Themed meetings

The meetings take place over a period of about six months, during which time groups of 8–12 people meet on five occasions for two or three hours at a time. The meetings are led by two persons whose main task is to arouse and deepen your thoughts concerning what palliative care is and how it is carried out at your workplace.

For each theme there is a short text accompanied by suggestions for discussion subjects, literature and links for use at the meetings. There are also tasks to be performed in preparation for the meetings. It is important that you go through the material in advance because the discussion will be more rewarding if there is a shared point of departure. The idea is that what emerges at the meetings shall be relevant to your daily work and that you shall be able to put it to practical use. In this respect it is important that you shall feel free to put forward your own questions for discussion.

The proper functioning of the meetings requires that all the participants are active and help create a positive and free atmosphere. It is important to remember that these meetings have nothing to do with *instruction* — it is not a question of the leader’s lecturing the group and feeding them with knowledge, but of the group’s taking up an issue within palliative care and the leader’s guiding and stimulating the discussion of it. All who take part have the right — or rather, the *obligation* — to prepare, to focus on the theme and to share their knowledge, experience, thoughts and ideas.

In connection with each theme you will be provided with suggestions as to areas for improvement and as to how you yourself can contribute to making things better. A simple plan of action can be of help here, and an example of such a plan is presented.

# Material for the meetings

As mentioned above, the material is based mainly on the WHO 2004, WHO 2011, NCP and the NKS (the two latter only in Swedish). These documents complement each other and can together serve as a basis for dignified palliative care. The documents themselves would be complemented with other literature relevant to the particular theme. In addition the group can themselves include other literature which is relevant in the specific context.

In both form and content the material derives from a collaboration involving patients, next of kin and staff, inspired by the learning plans on the theme of palliative care published by the Swedish Institute for Health Sciences. In the material we have chosen to use the term “patient”. This designation is to be regarded as synonymous with such other terms (e.g. “user”, “care recipient”) as are employed to refer to persons receiving care within the framework of health care.

We wish you inspiring and rewarding discussions which will contribute to the enhancement of palliative care both for the patients and from the point of view of the next of kin!

# The work of improvement

One purpose of the themed meetings is to stimulate the work of everyday improvement. The basic prerequisites for bringing about such improvement are having the will, having ideas and having the determination to follow through on these ideas. You also need to understand the system within which you are working, and to know about different methods and instruments. Below we describe one common useful way to work with of everyday improvement.

## The improvement model — a basic tool in the work of improvement

Set up a goal for what you want to achieve. Do a measurement to see whether a change corresponds to an improvement. Test the ideas for improvement one small step at a time. Draw up a plan of action and decide who is to do what. Work systematically, step by step.

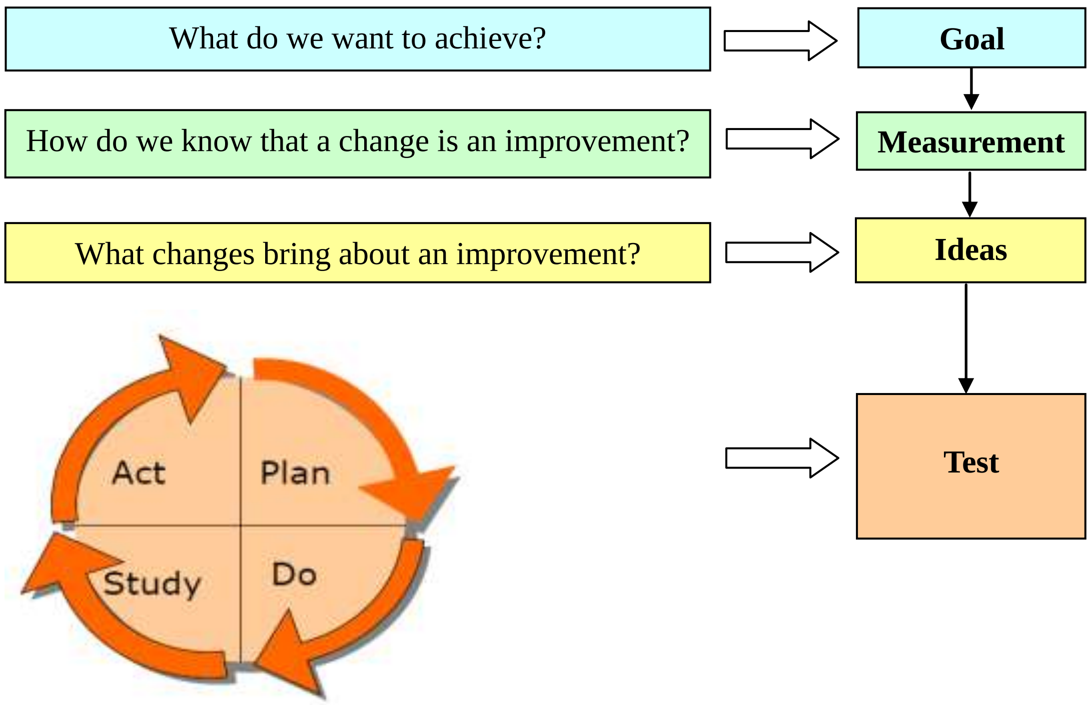


**Figure 1. Improvement model PDSA-circle**

## Plan of action

The use of a plan of action gives a clear structure to the work of improvement. Below is a suggestion as to what a plan of action might look like.

| Plan of action for... | | | |
|---|---|---|---|
| What's the problem? | What's causing it? | What's to be done about it? (Who by? When?) | Follow-up of the action |
| | | | |
| | | | |
| | | | |

# Selected references in English


Bathalden P. *What are a microsystem?* https://clinicalmicrosystem.org/ (2016-07-26)

Godfrey M.M. *Microsystem Academy*. http://tdi.dartmouth.edu/research/intervention/microsystem-academy

Nelson E.C., Batalden P.B. & Godfrey M.M. (2009). *Quality By Design*. San Francisco, CA: Jossey-Bass.

NHS (Institute for innovation and Improvement). *Quality and Service Improvements Tools: Plan, Study, Do Act (PDSA).* http://www.institute.nhs.uk/quality_and_service_improvement_tools/quality_and_service_improvement_tools/plan_do_study_act.html

Moss F., Palmberg M. Plsek P. & Schellekens w. (2000). Quality improvement around the world: how much we can learn from each other. Quality in Health Care, 9, pp. 63-66.

# The palliative approach and dignified care

**The purpose** of this theme is to increase the knowledge and understanding of the values on which palliative care is based, its ethics and its manner of encountering the patient.

**How to prepare for the theme meeting**
Think about how you look upon palliative care and write down a few points concerning what you see as being specific to palliative care.

## Palliative care

When it becomes impossible to restore the physical and mental health of a person who is gravely ill there is still a lot that can be done to make the person's quality of life as good as possible.

Palliative care is directed towards the alleviation of suffering and the enhancement of quality of life and dignity during the time the person has left, irrespective of age and diagnosis, without either shortening or lengthening life. The World Health Organisation has emphasised that everyone has the right to good palliative care, whatever the way in which this care is carried out or organised (WHO 2010, Wpca and WHO 2014).

*Discussion question:*

- Does this description of palliative care reflect your own experience with patients?

# The WHO definition of palliative care:

Palliative care is an approach that improves the quality of life of patients and their families facing the problem associated with life-threatening illness, through the prevention and relief of suffering by means of early identification and impeccable assessment and treatment of pain and other problems, physical, psychosocial and spiritual. Palliative care:

- provides relief from pain and other distressing symptoms;
- affirms life and regards dying as a normal process;
- intends neither to hasten or postpone death;
- integrates the psychological and spiritual aspects of patient care;
- offers a support system to help patients live as actively as possible until death;
- offers a support system to help the family cope during the patients illness and in their own bereavement;
- uses a team approach to address the needs of patients and their families, including bereavement counselling, if indicated;
- will enhance quality of life, and may also positively influence the course of illness;
- is applicable early in the course of illness, in conjunction with other therapies that are intended to prolong life, such as chemotherapy or radiation therapy, and includes those investigations needed to better understand and manage distressing clinical complications (WHO 2010).

*Discussion questions:*

- Discuss this definition of palliative care.
- What are its good and its less good points?

# The values underlying palliative care

From the WHO definition (WHO 2010) there emerge the following four cornerstones of palliative care. The first of them is the alleviation of symptoms (involving not only the physical aspect but also the mental, social and existential/spiritual aspects). The second cornerstone is inter-professional team-work, steered by the particular patient's needs. The third is continuity of care and good communication between patient, next of kin and staff, irrespective of where the leadership resides. The fourth and last cornerstone is the provision of adequate support for next of kin — for it is often the latter who make the basic contribution to care in life's final phase.

The values underlying palliative care can be summed up in four key terms: closeness, wholeness, knowledge and empathy.

It is in life’s final phase — as in life’s initial phase — that we are most vulnerable and most dependent on being taken care of by others. The distinguishing features of palliative care are its purpose and the extreme vulnerability of the patient. Which is to say that the purpose is not to combat illness but to help the patient to achieve the best possible quality of life, by means of alleviating symptoms and providing existential support in life’s final phase.

The Swedish National Board of Health and Welfare defines the palliative approach as an approach characterised by a holistic view of humankind whereby the individual is given support for living with dignity and the greatest possible wellbeing right up to the end.

*Discussion questions:*

- What parts of the palliative approach do you clearly apply in your work?
- What changes are needed at your workplace from the point of view of the application of a palliative approach?

## The transition to palliative care

Palliative care can be viewed from the perspective of two phases — the early and the late. The early phase can last a long time, whereby palliative care is given at an early stage of the illness in conjunction with life-extending treatment. The late phase, on the other hand, is short, and the whole purpose of care is to alleviate suffering and enhance quality of life (also in respect of the next of kin). The transition from the early to the late phase can be difficult to determine, and the borderline is often felt to be indistinct. In the case of certain diagnoses it can be difficult to distinguish a breakpoint between curative and palliative care. Both types of care can indeed be given during the same period. The basic transition process can be seen in terms of a change of main focus: cure→ life-extension→ alleviation.

A large majority of patients and close family members want to discuss end-of-life issues with their physician. Most of them expect their physician to initiate such dialogue. End-of-life discussions, however, must go beyond the narrow focus of resuscitation. Instead, such discussions should address the broad array of concerns shared by most dying patients and families: fears about dying, understanding prognosis, achieving important end-of-life goals, and attending to physical needs. Good communication can facilitate the development of a comprehensive treatment plan that is medically sound and concordant with the patient's wishes and values (Balaban 2000).

In the case of many elderly people with multiple illnesses or whose age is extremely high, the approach to life's final phase can be long-drawn-out, and it can be difficult to pinpoint when care should take a new direction. However this may be, sick elderly people are often in need of palliative care. In this connection it is important to note that there can occur several watersheds in the course of an illness, at each of which there should be a "break-point conversation" or end-of-life conversations. Thus there are often several such talks during the course of an illness.

The Swedish National Board of Health and Welfare defines the end-of-life conversations in conjunction with the transition to palliative care in life's final phase as a talk between doctor and patient concerning this transition and the nature of future care, from the perspective of the patient's condition, needs and wishes.

*Discussion questions:*

- How is the transition to palliative care handled at your workplace?
- How can next of kin become more involved in this process?

# Dignified care — preservation of the patient's dignity

In connection with palliative care it is important that the patient's dignity and self-image should be safeguarded and that the patient should preserve a sense of autonomy and control within a social framework. In the Swedish NCP 2012-2014 it is stated that all care should be imbued with respect for the individual's human worth and integrity. This means that patients have the right to respect for their identity and their body and also for their values, conceptions, outlook and faith. Respect for the human being must always be preserved, even after death.

The meaning of dignity is often taken to be self-evident but sometimes reference is made to different *types* of dignity. Two of these types are considered as being particularly relevant to care in life's final phase (Lam 2007). One of them is *human* dignity, which applies to all of us in that we are human beings and which gives us the right to equal respect and dignity. The other is dignity *of identity*, which is bound up with the human being as a person and concerns the person's integrity and autonomy. Identity can be threatened and infringed upon by e.g. illness, ageing and the attitude of those around. Identity can also be infringed upon after a person's death, e.g. through the manner in which the body is handled.

*Discussion question:*

- What's said about dignity at your workplace?

# Dignified care — a case history

*Stina is on the cancer ward and is dying. She wants her family with her but is very weak and is not sure that she can cope with having them there. Her daughter and members of the staff talk to her about the best way to arrange a good ending. There is an openness about the conversation from which it clearly emerges that all are aware that Stina's life will soon be over. Together they decide to inform the closest family that Stina wants them to come and be with her but that they must be prepared for the possibility of her not having the strength for it. Her sister, her brother, her daughter and the daughter's husband and their two small children spend a couple of hours with Stina. They bring coffee and cakes and sit chatting to Stina. The youngest grandchild sits curled up in bed with Granny. Members of staff discreetly enter at regular intervals, render pain relief, fluff up the pillows and discreetly leave. When Stina starts to get tired she feels it's quite OK to say so, and everyone except her daughter goes home. The daughter stays all night, and when Stina wakes up now and then they have a little chat. Stina becomes more and more difficult to maintain any contact with, and early in the morning she dies.*

*Discussion questions:*

- Briefly describe a situation where the patient's dignity was preserved.
- What ways do you have for preserving a patient's dignity in various situations?

# Non-dignified care — a case history

(Maria, Daniel's wife, recounts his last days:)
*Daniel was in the army and he's always been an imposing and well-dressed man. He's always been particular about his appearance and it's been a point of honour with him to act as a perfect gentleman. That's why it was so woeful for me to see the state he was in when I went to see him at the hospital. It would have been woeful for him too if he'd been aware of how things were — or perhaps he was aware of it, and was suffering without my noticing it. There he was, like a baby bird, curled up tight and freezing. He didn't even have a blanket over him, and there was nothing on his feet. No trousers — just net pants with a nappy in and a thin nightshirt. It was so demeaning.*

*Discussion question:*

- Have you had a situation where a patient's human dignity or dignity of identity was infringed on? If so, what could you have done differently?

## As follow-up...

Choose two of the items listed in the following section about *The rights of the dying* and discuss with the person in charge and your fellow-workers how these items are handled at your workplace and what, if any, improvements are needed.

## The rights of the dying

In discussing the palliative approach it is important to bear in mind the Dying Person's Bill of Rights, drawn up at a workshop in Lansing, Michigan, in 1975 and published in the *American Journal of Nursing* the same year. It reads as follows:

### The Dying Person's Bill of Rights

- I have the right to be treated as a living human being until I die.
- I have the right to maintain a sense of hopefulness however changing its focus may me.
- I have the right to be cared for by those who can maintain a sense of hopefulness, however changing this might be.
- I have the right to express my feelings and emotions about my approaching death in my own way.
- I have the right to participate in decision concerning my care.
- I have the right to expect continuing medical and nursing attention even though "cure" goals must be changed to comfort goals.
- I have the right not to die alone.
- I have the right to be free from pain.
- I have the right to have my questions answered honestly.
- I have the right not to be deceived.
- I have the right to have help from and for my family in accepting my death.
- I have the right to die in peace and dignity.
- I have the right to retain my individuality and not be judged for my decisions which may be contrary to beliefs of others.
- I have the right to discuss and enlarge my religious and/or spiritual experiences, whatever these may mean to others.
- I have the right to expect that the sanctity of the human body will be respected after death.
- I have the right to be cared for by caring, sensitive, knowledgeable people who will attempt to understand my needs and will be able to gain some satisfaction in helping me face my death (Barbus, 1975).

**References** (add relevant references to this theme in your countries language)

……………………

## Suggestions for further reading

………………

# Next of kin

**The purpose** of this theme is to increase knowledge and understanding of the situation and role of the next of kin and to consider how their need of support can better be met.

## How to prepare for the theme meeting

Choose from the following:

- Ask the next of kin of a person who is dying how their everyday life, and the everyday life of others close to the person, has been affected.
- Ask such a next of kin what is involved in being a participant in care and what importance it has for them.
- Ask them what support from care staff is of most importance, and why.
- Get in touch with a users' organisation (patients or next of kin) and ask them how they look upon the next of kin's participation in care, responsibility and need of support.

Be ready to bring up what you learn from this in the meeting with the colleagues.

## The situation of the next of kin

Life changes drastically when a member of the family or other loved-one contracts an incurable disease and can be expected to die within the near future. There is a sharp change in both the patient's and the next of kin's perspective on their individual lives and their life together. Two types of preparation are involved: on the one hand there is the patient's preparation for dying, on the other the next of kin's preparation for going on living after the death of a loved-one. The next of kin's need for support can vary from one stage of the illness to another. It is important that all of the next of kin's needs should be taken into account — on the one hand their needs in the role of informal carer which they have assumed or which has been conferred on them, on the other their own personal needs (Andershed 2006).

Being a person's next of kin does not automatically imply a biological relationship — the next of kin can be a friend or neighbour, for instance. But whatever their relationship to the sick person may be, and however old they may be, and whatever their cultural origins may be, the next of kin will be affected by the progress of the illness and the confrontation with death and dying.

*Discussion question:*

- How do you look upon the notion of next of kin and that of family? What is their significance in relation to care?

# Children as next of kin

Children, too, need to understand what is happening, need to play a part in it and need to have access to support. In 2010 an amendment was made to the Swedish Health Care Act for the purpose of reinforcing children' rights as next of kin, whereby it was laid down that:

§2 …. Healthcare staff shall pay special regard to a child's need of information, counsel and support if the child's parent (or other person with whom the child is living) has a serious physical illness or unexpectedly dies.

In order that this shall be complied with it is necessary to determine whether there are routines in place for (a) asking and journalising whether or not there are children in the family and (b) asking the parent(s)/guardian what the children have been told about the illness and the prognosis.

Children need to receive information about (Hospice Friendly Hospital Programme's 2016)

- the illness (cause, care, treatment, the serious prognosis)
- how the illness can affect themselves and their parent(s)/guardian both before and after the time of death (thoughts, feelings, practical matters)
- who they can turn to if they want to ask questions or express their thoughts

*Discussion questions:*

- How are children's needs as next of kin taken into account at your workplace?
- Is there room for improvement?

# Participation

The next of kin share much of the responsibility for the care, especially if the care is given at home. Despite the fact that taking care of the patient can be trying and tiring, the next of kin for the most part express satisfaction with their task. It can happen, though, that next of kin feel themselves *reduced to* their role of informal carer, at the expense of their normal role vis-à-vis the patient (as e.g. spouse or child). Though it is common for next of kin to want to be close to the sick person (which can include participation in the care of this person), there are exceptions. The next of kin’s participation in the care has been described as taking place either “in the dark” or “in the light”. The first of these two characterisations implies a lack of information and a sense of having to grope one’s way forward, the second implies information, trust, respect and meaningfulness. For next of kin to be able to feel that they are making a tangible and positive contribution there must be a constant sharing of comprehensible information. Participation can contribute to the next of kin’s recovery after the person’s death (Andershed & Ternestedt 2001, Andershed 2006, Wallerstedt et al. 2014).

*Discussion question:*

- In what way do next of kin participate in care at your workplace?

# Support

During the period of care the next of kin can experience a sense of insufficiency, powerlessness and helplessness, and they may have to deal with existential questions. They can also feel shame and guilt. The next of kin’s need for support can vary and can be altogether different from that of the patient, so the support has to be adapted to each person’s particular needs. Support can be either emotional (in the form of dialogue, for instance) or practical (in the form of respite or benefit, for instance). It is laid down in the Swedish Social Services Act that next of kin who are taking care of a chronically sick or elderly person shall be given support and that their task shall be facilitated. The purpose of such support is to prevent the next of kin’s falling ill and to make them more able to cope with the difficulties arising during the period of care and with their grief after the person has died (Roulston et al. 2016, Andershed & Ternestedt 2001, Andershed 2006, Wallerstedt et al. 2014

Next of kin can begin their mourning during the care period (“anticipatory mourning”). It is important to bear in mind that mourning can be expressed in many ways and has different phases (Clukey 2008).

In the definition offered by the Swedish National Board of Health and Welfare a *post-decease conversation* (or “survivor conversation”) is a talk between a member of the care

staff and the next of kin of the deceased person some time after death has occurred. This conversation gives the next of kin the chance to offer views on the care and to round off the relationship with the staff, at the same time as it gives the staff the chance to benefit from these views when it comes to improvement of the quality of care.

*Discussion questions:*

- How do you keep account of the next of kin's needs and what support is offered?
- Are post-decease conversation offered?

# As follow-up...

The Swedish palliative register list several quality indicators. Read the article and select quality indicators that are relevant to the situation of the next of kin (Lind et al. 2013, Lundström et al. 2012). Discuss with colleagues what improvements might be made in this respect at your workplace.

**Post-decease conversations**

- Find out whether such talks are offered where you work.
- If they are, how do you put the information to use?
  If they aren't, what do you need to do to change this?
- In co-operation with the person in charge and those you work most closely with, draw up a simple plan of action concerning how such talks are to be conducted.

**Children as next of kin**

- Find out what account is taken of children as next of kin where you work.
- What routines are there for asking and journalising whether there are children in the family?
- What routines are there for asking parents/guardians what the children have been told about the illness and the prognosis?
- What support is there for children as next of kin?
- In co-operation with the person in charge and those you work most closely with, draw up a simple plan of action concerning children as next of kin.

## **References** (add relevant references to this theme in your countries language)

……………………

## Suggestions for further reading

………………

# Existence and dying

**The purpose** of this theme is to increase your awareness and understanding of your own thoughts concerning existence and death with dignity, this in order that you shall feel secure in difficult conversations with patients and next of kin.

**How to prepare for the theme meeting**

Listen to a person, an expert (for example a priest or a psychologist) with extensive experience in the field, who talks about existence and dying.

Write down three reflections you got when listen to the programme. Share your thoughts and ideas with the colleagues in the meeting.

None can flee from death. It follows life, as surely as night follows day and as winter follows autumn. When death can no longer be escaped and life is drawing to its end, the human being is often confronted with physical, social and existential challenges and difficulties. In contrast with death, which is universal, life's final phase is individual, whereby we all have our own unique experiences, wishes and needs (text from the NCP).

## Existence

A great challenge confronting palliative care staff is talking to patients about death and dying. Quite often there is an avoidance of such existential questions as the following: Why are we here on earth? Why do we have to die? What's life's meaning? Why is there pain, suffering and death? Where do joy, hope, happiness and love come from? Is there some good higher Being, a God who cares about us?

*Discussion question:*

- What are your own thoughts when it comes to existential questions, spirituality and faith?

Existential questions often come to the fore in border situations, which is to say in situations where we are forced to make choices that will have a marked effect on the rest of our lives. Such situations can be positive but can also be threatening or distressing. The type of border

situation where death comes into view can bring about an existential crisis on the part of both patient and next of kin. Often there are no evident answers to existential questions, for which reason it can feel difficult to talk about them. Staff can be worried that such talk might arouse feelings they are unable to handle or questions they are unable to answer. It is therefore a great advantage if staff can discover strategies for feeling more secure in discussing existential questions with patients and next of kin.

**Suggestions as to starting-points for the discussion of existential questions**

- What question would you most like to have answered right now?
- What question is most urgent for you?
- What are you thinking about right now?
- What would happen if... ?
- What do you think... ?

It is at the same time important to bear in mind that there are patients and next of kin who do not want to talk about such questions, also that sometimes there can be communication without words. What you can do as a member of staff is to provide the opportunity for conversation, to *be there* — attentive, listening, ready to gently guide the conversation. A patient experiencing an existential crisis needs a sense of security as a basis for grappling with it in his/her own way and at his/her own speed, and needs help in understanding it, finding sense in it and surviving it. There can arise a special understanding between patient and staff, involving the sharing of something — a knowledge of illness and its course — which cannot be shared in the same way with next of kin. And such an understanding paves the way for talking about existential questions.

*Discussion questions:*

- Do you feel that you can take up death, dying and existential questions with patients and their next of kin? If the answer is yes, *why* do you feel that you can? If the answer is no, *why* do you feel that you can't?
- What could be done at your workplace to make it easier to talk about existential questions and loneliness?

# Dying

The dying person gradually loses all capacities, and the basic care needs must be met and the person's wishes respected. It is difficult to predict just how much time a person has left. For want of greater precision, we can think in terms of death's being "inevitable within the foreseeable future", by which is meant that it is a question of a few hours, days, weeks or months.

*Discussion question:*

- How do you yourself look on death? What do you think happens?

Dying with dignity implies having one's human dignity recognised, being taken care of with respect and empathy, being listened to and heeded, having one's physical and emotional suffering alleviated, having one's privacy guaranteed, being able to maintain emotional ties and sort out personal matters, and having access to spiritual or existential support. The preservation of dignity is not only a question of the care which the patient receives but also of the way in which this care is given, of the carer's approach and general behaviour (Proulx & Jacelon 2004).

*Discussion question:*

- What, in your work, has contributed to making death, *death with dignity*?

# The place where care is given

Research has indicated that when healthy people are asked where they would like to die, the majority state that they would like to die at home. When sick people are asked the same question, however, their answers can change over time. Despite the importance of where we spend life's final phase, the dying person is not always invited to take part in deciding where it shall be.

One of the goals of palliative care is to foster a sense of belonging, this in the sense that wherever the care is given (e.g. hospital, home for the elderly), the patient shall feel at home. Here the quality of the care is an important factor. The patient's sense of identity, sense of being seen as an individual, depends on the patient–staff relationship. If this relationship is good and one as a patient can be oneself, on one's own conditions, one has a better chance of focusing on life here and now rather than on the approach of death. Respect for the private room, for the private space, implies the creation of a private sphere. Two important aspects of this are that a closed door should be respected by staff and that one should have the chance to

pursue in privacy such activities as one pursued where one used to live. The creation of emotional ties to the place where one now finds oneself can be fostered by the presence of personal belongings and by there being public rooms which are inviting and hospitable.

**The feeling of being at home depends on:**

- Being able to be oneself and being seen as a person
- Respect for the private room and the private space
- The creation of emotional ties to the place

The sense of belonging is established over time and by means of activities, which calls for continuity of care and the development of the patient–staff relationship (Barbus 1975, Proulx & Jacelon 2004, WHO 2010, Wpca and WHO 2014).

*Discussion questions:*

- In what way can we as staff facilitate the patient's having a say in where they want to die?
- What do we do at our workplace to see that patients feel at home?

# The dead body

Respect shown for death and the dead body is an extension of respect shown for human integrity. It is also a manner of showing consideration for those left behind and their feelings (Wilkinson 2014). How the dead person is to be treated is a broadly human question. Some people feel fear when confronted with a dead body and do not like the idea of having a dead body in their home. Such fear is understandable.

*Discussion question:*

- How can we as staff offer support to next of kin who feel fear and discomfort when confronted with the dead body?

It is important to be aware that there are regulations concerning how the dead body is to be taken care of, and that these regulations imply respect for the dead person. In the Swedish

Health Services Act it is stated: “When someone has died, the tasks of public health and sick care shall be performed with respect for the deceased. Respect and consideration shall be shown to those left behind.” There are also cultural rules governing how the dead person is to be taken care of. Nowadays we who work in the sphere of care encounter people from a variety of cultures with a variety of traditions, and this calls for knowledge and openness.

*Discussion question:*

- How do we show respect for the deceased and consideration for the persons left behind?

## As follow-up...

To learn more ……

- Think about how you yourself can make it easier to talk about existential questions.
- Discuss with the person in charge and your colleagues what can be done at your workplace to facilitate the discussion of existential questions.
- Draw up a plan of action with regard to what can be done at your workplace to bring existential questions to the fore.

## References (add relevant references to this theme in your countries language)

……………………

## Suggestions for further reading

……………….

# The alleviation of symptoms

**The purpose** of this theme is to increase understanding of the importance of good alleviation of symptoms in palliative care and to promote the use of validated rating instruments.

### What to do to prepare for the theme meeting

- Talk to a patient about their symptoms. Think about how the symptoms are expressed and about how we as staff can take account of them. Be ready to convey your thoughts to others at the meeting.
- Familiarise yourself with how symptoms are alleviated at your unit (which symptoms, how often, with what? etc.).
- If you are acquainted with any instruments for the assessment of symptoms, get ready to present them at the meeting, so that they can be discussed in respect of possible utilisation at your workplace.

## Symptoms

Alleviation of symptoms and the fostering of wellbeing are important tasks in the care of the gravely ill and dying. Through the forestalling, early discovery and management of different sorts of problems both the patient and the next of kin can be given the possibility of the best possible quality of life and wellbeing during life's final phase.

*Discussion questions:*

- Talk to one another about your conversations with patients on the subject of their experiences of symptoms
- How does this relate to what you found out about how symptoms alleviation work at your unit?

Bear in mind that symptoms can be physical, mental, social and existential.

The question of care and treatment directed towards the alleviation of suffering and the increase of wellbeing is taken up in the WHO 2011 (Hall et al. 2011). There is a description of some symptoms —and of how they can be alleviated. Symptoms can be purely physical in origin but can also have non-physical origins such as unresolved conflicts.

*Discussion question:*

- How can you take account of the patient's symptoms?

## The assessment of symptoms

In the Swedish NKS it is recommended that staff in health care and the social services should perform regular analysis and structured evaluation of the patient's symptoms for the purpose of achieving the best possible symptom alleviation.

## Examples of instruments for the assessment of symptoms

- ESAS — the Edmonton Symptom Assessment Scale, a 10-point scale for assessing nine common symptoms
- NRS — the Numeric Rating Scale, a 10-point scale for assessing pain and certain other symptoms
- VAS — the Visual Analog Scale, a 10-point scale for assessing pain and certain other symptoms
- the Abbey Pain Scale, for assessing pain in the case of people who cannot verbalise
- the Norton Scale, for assessing the risk of pressure ulcers
- the Downton Fall Risk Index, for identifying the risk of falling
- ROAG — the Revised Oral Assessment Guide, for assessing oral health
- MNA — the Mini Nutritional Assessment, designed to screen for nutritional problems

# General symptom assessment

The Edmonton Symptom Assessment Scale (ESAS) covers the following: pain, tiredness, nausea, depression, anxiety, drowsiness, appetite, wellbeing and shortness of breath. Each of these nine factors is rated on a scale of 0–10, where 0 means that the symptom is absent (or that there is appetite or wellbeing) and 10 means that there is the worst possible severity (or worst possible appetite or wellbeing). It is recommended that ESAS be used at the first meeting with a patient. Thereafter it can be used once a week and/or in the case of need (Capital Health & Caritas Health Group 2005).

# Specific symptom assessment

Pain (and certain other symptoms) can be rated on the 10-point Numeric Rating Scale (NRS) or Visual Analog Scale (VAS). Though these scales have the advantage of being easy to use, a drawback is that they only measure degree of intensity. Furthermore they require the active participation of the patient, which means that they cannot be used in the case of cognitive incapacity (e.g. dementia) or other such incapacity as impedes communication. In such cases other instruments can be used. A good example of these is the Abbey Pain Scale (Abbey 2004). The assessment is based on six questions about the person's way of expressing themselves, bodily functions and behaviour. Each question is scored on a 4-point scale (Absent 0, Mild 1, Moderate 2, Severe 3) and the six scores are added up. The total scores are graded as follows: 0–2 no pain, 3–7 mild pain, 8–13 moderate pain, 14+ severe pain.

*Discussion questions:*

- What rating instruments have you brought with you, or had experience of?
- When can data and rating instruments be of help?
- How can you use ESAS, NRS and the Abbey Pain Scale?

# The alleviation of symptoms

There is comprehensive descriptions of the alleviation of specific symptoms in the literature, therefore we do not take it up here but instead recommend that you read a book in palliative medicine (for example Geoffrey 2010).

*Discussion questions:*

- Choose two or three symptoms and discuss how you at present assess them and alleviate them. Then compare your procedures with those recommended in palliative medicine. Are there any differences? Is there anything that you need to change?

- How do you evaluate the alleviation of symptoms?

Bear in mind that palliative care in life’s final phase is *active* care and treatment.

## As follow-up...

Draw up a simple plan of action (see page 7-8) on the basis of what improvements are needed at your workplace with regard to the alleviation of symptoms. Get your fellow-workers involved in this.

## References ((add relevant references to this theme in your countries language)

……………………

## Suggestions for further reading

………………

# Collaborative care

**The purpose** of this theme is to bring about an increased understanding of the importance of teamwork, with particular focus on care as implying collaboration between staff, patient and next of kin.

## How to prepare for the theme meeting

Write down your thoughts about what collaborative care *is*. Think about what are the prerequisites for care as collaboration between staff, patients and next of kin. What are the conditions which favour such collaboration? And what are those which stand in its way?

The Swedish news anchor Ulla-Carin Lindquist, who died in 2004, was diagnosed as having ALS and she had difficulty swallowing. The following is from the book she wrote about her illness:

*"They called from the hospital," says the personal assistant. "You're going to have a hole made in your stomach on Tuesday. I talked to your doctor too."*
*"On Tuesday! It was supposed to be a week from now."*
*"They think it won't wait. You don't weigh much over seven and a half stone now."*
*"Why didn't they want to talk to me? Why don't they inform me?"*
*"They thought I could tell you... "*
*"Bloody hell!"*

*Discussion question:*

- What thoughts come into your mind when you read this?

## The concept of collaborative care

The basis for collaboration in care is the joint creation of knowledge. In a care situation there occurs a daily exchange of knowledge, both formally and informally. From the encounter between different sorts of experiences new knowledge is created. In the light of this it is

natural that patients and next of kin should participate in the decision-making with staff. A prerequisite for this collaboration is that both parties — patients and next of kin on the one hand, staff on the other — have the will to function as a team, in a genuine partnership where the two parties have the same worth.

*Discussion question:*

- Share with one another your thoughts about what collaborative care is and about what favourable conditions exist for it and what might stand in its way.

# Participation and self-determination

In a number of official documents in the sphere of healthcare it is emphasised how important it is that the patient should be seen as a person and that the care should be adapted to the needs and capacity of the individual. It is also emphasised that patients and next of kin should be given the opportunity to participate in the decision-making concerning the care in question. The starting-point for collaborative care is the situation of the particular patient and the extent to which the patient and the next of kin want to participate.

The purpose of the Swedish Patient Act which came into force on 1 January 2015 is to strengthen and clarify the patient's status and to promote the patient's integrity, self-determination and participation.

*Discussion question:*

- How can you promote the patient's integrity, self-determination and participation?

Information about the patient's state of health and the content of care is a prerequisite for participation and collaboration. But if the patient and the next of kin are to be able to give their *informed consent* it is important that staff do not simply provide information from their own perspective but also — indeed, *principally* — provide such information as the patient and the next of kin are asking for. This means that special demands will be put on staff if the patient's autonomy is limited for one reason or another or if an interpreter is needed.

*Discussion questions:*

- What do you do to enable the participation of patients and next of kin in the care?
- How do you ascertain to what extent they want to participate?

- How can you make it possible for vulnerable persons (e.g. those with reduced autonomy or who have language difficulties) to participate?

# The prerequisites for collaborative care

Palliative care is complex and requires the collaboration of patient, next of kin and staff. The care is provided both formally, by professional staff, and (to an increasing extent) informally, by next of kin. This calls for teamwork. The concept of teamwork is often used with reference to collaboration within and between groups of professionals, but it is applicable also to collaboration between patients, next of kin and staff. Collaboration in care promotes the best possible quality of life both for the patient and for the next of kin. Furthermore, studies have indicated that such collaboration has a beneficial effect on the wellbeing of staff as well, and can lead to lasting improvements in care.

*Discussion question:*

- How do you utilise the competence of patients, next of kin and staff in care?

# The act of collaboration

One approach to collaboration in care is to set in motion an open and constructive dialogue, where the patient and the next of kin are seen as competent team-members and where different professional groups may participate. Thus teamwork can be used as an instrument for achieving collaborative care. A team has been defined as *a group of people who work in the vicinity of one another, who collaborate and co-ordinate their efforts and who, in a general sense, work towards the same goal*. In order that the collaboration and co-ordination shall be optimal it is necessary that there should be well-functioning communication within the team and that there should no sense of hierarchy. An open dialogue on equal terms increases the chances of establishing trust — trust which enables people’s ideas, hopes and fears to come into the open and which fosters mutual understanding.

*Discussion questions:*

- What is your teamwork like, with patients and next of kin and members of other groups of professionals?
- What importance do you think teamwork has for patients and next of kin?

# As follow-up...

Discuss collaborative care with your fellow-workers and the person in charge, on the basis of the following questions:

- What more can be done at your workplace to ensure the teamwork that forms the basis for collaborative care?
- What works well at present and what requires greater attention?

# References ((add relevant references to this theme in your countries language)

……………………

# Suggestions for further reading

………………

# References in English

Abbey J, Piller N, De Bellis A, Esterman A, Parker D, Giles L. & Lowcay B. (2004). *The Abbey pain scale: a 1-minute numerical indicator for people with end-stage dementia. International Journal of Palliative Nursing* 2004;10(1):6-13.

Andershed B. & Ternestedt B-M. Development of a theoretical framework describing relatives' involvement in palliative care. *Journal of Advanced Nursing* 2001; 34(4): 554-562.

Andershed B. Relatives in end-of-life care - part 1: a systematic review of the literature the five last years. *Journal of Clinical Nursing* 2006;15(9):1158-1169.

American Academy of Hospice and Palliative Medicine. (2016). *Frequently Asked Questions About Hospice and Palliative Care*. Chigao: http://palliativedoctors.org/faq

Balaban R,B. A Physician's Guide to Talking About End-of-Life Care. *Journal of General Internal Medicine* 2000 Mar; 15(3): 195–200. doi: 10.1046/j.1525-1497.2000.07228.x'

Barbus A.J. The Dying Person's Bill of Rights. *The American Journal of Nursing* 1975; 75 (1): p. 99. http://www.jstor.org/stable/3423093

Capital Health & Caritas Health Group. (2005). Edmonton Symptom Assessment System Graph (ESAS). 2016-07-26.
http://www.npcrc.org/files/news/edmonton_symptom_assessment_scale.pdf

Clukey L. Anticipatory mourning: processes of expected loss in palliative care. *International Journal of Palliative Nursing* 2008;14(7): 316-325.

Davies E and Higginson I J. eds (2004). *Better Palliative Care for Older People*. WHO:
http://www.euro.who.int/__data/assets/pdf_file/0009/98235/E82933.pdf

EAPC (European Association of Palliative Care). (2016). *Definition of Palliative care*.
http://www.eapcnet.eu/corporate/abouttheeapc/definitionandaims.aspx

Geoffrey H, Nathan I.C, Nicholas A.C, Marie F, Kassa S, Russell K.P, eds. (2010). *Oxford Textbook of Palliative Medicine, Fourth Edition***.** Oxford, UK: Oxford University Press, 1-666 pp.

Hall S, Petkova H, Tsouros A.D, Costantini M. and Higginson I.J. (2011). *Palliative care for older people: better practices*. WHO Regional Office for Europe:
http://www.euro.who.int/__data/assets/pdf_file/0017/143153/e95052.pdf

Lam K.K, (2007). Dignity, Respect for Dignity, and Dignity Conserving in Palliative Care. Report Palliative Medical Unit, Grantham Hospital from Palliative Medicine Doctors' Meeting:
www.fmshk.org/database/articles/dignityrespectfordignityanddignityconservinginpall.pdf

Hospice Friendly Hospital Programme's. *Caring for a Dying Patient.* 2016-07-26.
http://hospicefoundation.ie/wp-content/uploads/2013/04/7.Caring-for-a-Dying-Patient.pdf

Lind S, Adolfsson J, Axelsson B. & Fürst CJ. Quality indicators for palliative and end of life care: a review of Swedish policy documents. BMJ Supportive & Palliative Care. 2013;3(2):174-80. doi: 10.1136/bmjspcare-2012-000390.

Lundström S, Axelsson B, Heedman PA, Fransson G & Furst CJ. Developing a national quality register in end-of-life care: The Swedish experience. *Palliative Medicine* 2012;26(4):313-321.

NHS (Institute for innovation and Improvement). *Quality and Service Improvements Tools: Plan, Study, Do Act (PDSA).* http://www.institute.nhs.uk/quality_and_service_improvement_tools/quality_and_service_improvement_tools/plan_do_study_act.html

Proulx K. & Jacelon C. Dying with dignity: the good patient versus the good death. *American Journal of Hospice & Palliative Medicine* 2004;21(2):116-120.

Roulston A, Campbell A, Cairnduff V, Fitzpatrick D, Donnelly C. & Gavin A. Bereavement outcomes: A quantitative survey identifying risk factors in informal carers bereaved through cancer. *Palliative Medicine* 2016; May 11. pii: 0269216316649127. [Epub ahead of print]

Wallerstedt B, Andershed B. & Benzein E. Family members' caregiving situations in palliative home care when sitting service is received: The understanding of multiple realities. Palliative Support Care 2014;12(6): 425-37. doi: 10.1017/S1478951513000333. Epub 2013 Jun 20.

Wilkinson TM. Respect for the dead and the ethics of anatomy. *Clinical Anatomy* 2014;27(3):286-90. doi: 10.1002/ca.22263.

WHO (2010). *Cancer: WHO Definition of Palliative Care*. http://www.who.int/cancer/palliative/definition/en/

Wpca and WHO. (2014). *Global Atlas of Palliative Care at the End of Life*. Wpca (worldwide palliative care alliance). http://www.who.int/nmh/Global_Atlas_of_Palliative_Care.pdf
